# Daratumumab plus lenalidomide and dexamethasone in relapsed/refractory multiple myeloma: extended follow-up of POLLUX, a randomized, open-label, phase 3 study

**DOI:** 10.1038/s41375-020-0711-6

**Published:** 2020-01-30

**Authors:** Nizar J. Bahlis, Meletios A. Dimopoulos, Darrell J. White, Lotfi Benboubker, Gordon Cook, Merav Leiba, P. Joy Ho, Kihyun Kim, Naoki Takezako, Philippe Moreau, Jonathan L. Kaufman, Maria Krevvata, Christopher Chiu, Xiang Qin, Linda Okonkwo, Sonali Trivedi, Jon Ukropec, Ming Qi, Jesus San-Miguel

**Affiliations:** 1 University of Calgary, Charbonneau Cancer Research Institute, Calgary, AB Canada; 2 The National and Kapodistrian University of Athens, Athens, Greece; 3 QEII Health Sciences Center and Dalhousie University, Halifax, NS Canada; 4 Service d’Hématologie et Thérapie Cellulaire, Hôpital Bretonneau, Centre Hospitalier Régional Universitaire (CHRU), Tours, France; 5 St James’s Institute of Oncology, Leeds Teaching Hospitals National Health Service Trust and University of Leeds, Leeds, UK; 6 Assuta University Hospital, Faculty of Health Science, Ben-Gurion University of the Negev, Beersheba, Israel; 7 Institute of Haematology, Royal Prince Alfred Hospital, Camperdown, NSW Australia; 8 Department of Medicine, Samsung Medical Center, Sungkyunkwan University School of Medicine, Seoul, South Korea; 9 Department of Hematology, National Hospital Organization Disaster Medical Center of Japan, Tachikawa, Japan; 10 Hematology, University Hospital Hôtel-Dieu, Nantes, France; 11 Winship Cancer Institute, Emory University, Atlanta, GA USA; 12 Janssen Research & Development, LLC, Spring House, PA USA; 13 Janssen Research & Development, LLC, Raritan, NJ USA; 14 Janssen Global Medical Affairs, Horsham, PA USA; 15 Clínica Universidad de Navarra-Centro de Investigación Médica Aplicada, Instituto de Investigación Sanitaria de Navarra, Centro de Investigación Biomédica en Red de Cáncer, Pamplona, Spain

**Keywords:** Cancer, Medical research

## Abstract

In POLLUX, daratumumab (D) plus lenalidomide/dexamethasone (Rd) reduced the risk of disease progression or death by 63% and increased the overall response rate (ORR) versus Rd in relapsed/refractory multiple myeloma (RRMM). Updated efficacy and safety after >3 years of follow-up are presented. Patients (*N* = 569) with ≥1 prior line received Rd (lenalidomide, 25 mg, on Days 1–21 of each 28-day cycle; dexamethasone, 40 mg, weekly) ± daratumumab at the approved dosing schedule. Minimal residual disease (MRD) was assessed by next-generation sequencing. After 44.3 months median follow-up, D-Rd prolonged progression-free survival (PFS) in the intent-to-treat population (median 44.5 vs 17.5 months; HR, 0.44; 95% CI, 0.35–0.55; *P* < 0.0001) and in patient subgroups. D-Rd demonstrated higher ORR (92.9 vs 76.4%; *P* < 0.0001) and deeper responses, including complete response or better (56.6 vs 23.2%; *P* < 0.0001) and MRD negativity (10^–5^; 30.4 vs 5.3%; *P* < 0.0001). Median time to next therapy was prolonged with D-Rd (50.6 vs 23.1 months; HR, 0.39; 95% CI, 0.31–0.50; *P* < 0.0001). Median PFS on subsequent line of therapy (PFS2) was not reached with D-Rd versus 31.7 months with Rd (HR, 0.53; 95% CI, 0.42–0.68; *P* < 0.0001). No new safety concerns were reported. These data support using D-Rd in patients with RRMM after first relapse.

## Introduction

The development of first- and second-generation novel agents over the past decade has led to the acceptance of immunomodulatory drug (IMiD)–based or proteasome inhibitor (PI)–based doublet or triplet therapy as standard of care for newly diagnosed and relapsed or refractory multiple myeloma (RRMM) [1, 2]. However, multiple myeloma (MM) remains an incurable disease, underscoring the need for new treatment strategies. Optimal combination and sequencing of these next-generation agents remain to be defined, particularly among various patient subgroups.

Daratumumab is a human immunoglobulin Gκ monoclonal antibody targeting CD38 with a direct on-tumor [3–6] and immunomodulatory mechanism of action [7–9]. Daratumumab-induced on-tumor activity occurs through several CD38 immune-mediated actions (complement-dependent cytotoxicity, antibody-dependent cellular cytotoxicity, and antibody-dependent cellular phagocytosis), apoptosis, and modulation of CD38 enzymatic activity [3–6]. The immunomodulatory actions of daratumumab minimize the immune-suppressive functions of CD38^+^ myeloid-derived tumor suppressor cells, regulatory T cells, and regulatory B cells and increase T-cell clonality [7–9].

Daratumumab has demonstrated single-agent activity in heavily pretreated RRMM and in combination with standard-of-care regimens in RRMM after at least one prior therapy [10–13]. Daratumumab safety and efficacy are also established in combination with bortezomib, melphalan, and prednisone, and with lenalidomide and dexamethasone in patients with newly diagnosed MM (NDMM) who are transplantation ineligible [14, 15].

In the prespecified interim analysis of the phase 3 POLLUX study (median follow-up, 13.5 months), daratumumab in combination with lenalidomide and dexamethasone (D-Rd) reduced the risk of disease progression or death by 63% (median progression-free survival [PFS] not reached vs 18.4 months; hazard ratio [HR], 0.37; 95% confidence interval [CI], 0.27–0.52; *P* *<* 0.001) and significantly increased the overall response rate (ORR) compared with lenalidomide and dexamethasone (Rd) alone (93 vs 76%; *P* *<* 0.001) in patients with at least one prior therapy [12]. In an updated analysis after a longer follow-up of 25.4 months, the PFS benefit of D-Rd was maintained compared with Rd, with median PFS still not being reached for D-Rd versus 17.5 months for Rd. In addition, deep and durable responses were achieved with D-Rd, and the PFS benefit of D-Rd versus Rd was consistently maintained regardless of the number of prior lines of therapy received, prior IMiD exposure, bortezomib refractoriness, time since last therapy, or cytogenetic risk [16].

We report the long-term efficacy and safety analyses of POLLUX after a median follow-up of more than 3.5 years.

## Subjects and methods

### Study design and patients

POLLUX is an ongoing, randomized, open-label, multicenter, phase 3 study in patients with RRMM (ClinicalTrials.gov Identifier: NCT02076009). An independent ethics committee or institutional review board at each site approved the trial, and all patients provided written informed consent. The study protocol was conducted in accordance with the principles of the Declaration of Helsinki and the International Conference on Harmonisation Good Clinical Practice guidelines. The study design, primary results, and post hoc secondary analyses have been previously reported [12, 16]. Briefly, eligible patients had progressive disease (according to International Myeloma Working Group [IMWG] criteria) [17, 18] during or after their last regimen, received and responded to at least one prior line of therapy, and had a creatinine clearance ≥30 mL/min. Prior lenalidomide exposure was allowed, but patients with lenalidomide-refractory disease were excluded from participation. A total of 569 patients were randomly assigned (1:1) using an interactive web response system to Rd (lenalidomide: 25 mg orally on Days 1–21 of each 28-day cycle; dexamethasone: 40 mg orally weekly) with or without daratumumab (16 mg/kg intravenous weekly for 8 weeks, every 2 weeks for 16 weeks, and every 4 weeks thereafter) until progression. The randomization was balanced by using randomly permuted blocks and was stratified by International Staging System (ISS), number of prior lines of therapy (1 vs 2 or 3 vs >3), and prior lenalidomide exposure. Treatment assignments were not blinded.

### Endpoints and assessments

The primary efficacy endpoint was PFS. Secondary efficacy endpoints included ORR, rates of very good partial response (VGPR) or better and complete response (CR) or better, minimal residual disease (MRD), time to response, duration of response, and overall survival (OS). PFS on subsequent line of therapy (PFS2) was an exploratory endpoint and was defined as the time from randomization to progression after the next line of subsequent therapy or death.

Exploratory post hoc secondary analyses evaluated patient subgroups according to prior lines of therapy (1 and 1–3), prior lenalidomide treatment, refractoriness to bortezomib, and achievement of CR or better. The number of prior lines of therapy was determined by investigators according to the IMWG consensus guidelines [18]. PFS, ORR, and MRD negativity were assessed for each subgroup.

MRD was assessed at the time of suspected CR and at 3 and 6 months after confirmed CR (and every 12 months thereafter if CR was maintained) using clonoSEQ® V2.0 (Adaptive Biotechnologies, Seattle, WA, USA). To allow for stringent, unbiased MRD evaluation, the entire intent-to-treat (ITT) population was evaluated, and patients were considered MRD positive if they had MRD-positive test results or no MRD assessment.

### Statistical analyses

Statistical analyses have been described previously [12]. Approximately 560 patients were randomized to observe 295 PFS events to detect an HR of 0.7 for the D-Rd group relative to the Rd group with 85% power at a two-sided significance level of 0.025, using a group sequential testing design.

PFS was compared between treatment groups based on a stratified log-rank test. HRs and 95% CIs were estimated using a stratified Cox regression model with treatment as the sole explanatory variable, and the Kaplan–Meier method was used to estimate the distributions. Stratified Cochran–Mantel–Haenszel tests were used to test treatment differences in ORRs and rates of VGPR or better and CR or better. MRD-negative rates were compared between groups using a Fisher’s exact test and the likelihood-ratio test.

## Results

Between June 16, 2014 and July 14, 2015, 569 patients at 135 sites in 18 countries across North America, Europe, and the Asia Pacific region were randomly assigned in POLLUX; 286 patients were assigned to D-Rd and 283 patients were assigned to Rd. Baseline patient demographics, prior treatment history, and other clinical and cytogenetic characteristics have been previously published [12, 16] and are summarized in Supplementary Table 1. Approximately half of patients (52%) had received one prior line of therapy, 18% had received prior lenalidomide, 44% had received prior IMiD and PI, and 21% were refractory to bortezomib.

At the clinical cutoff on October 10, 2018, a total of 158 (55.8%) patients in the D-Rd group and 237 (84.3%) patients in the Rd group had discontinued treatment. The most common reasons for discontinuation of treatment were progressive disease (D-Rd, 33.2%; Rd, 59.4%) and adverse events (AEs; D-Rd, 14.8%; Rd, 14.9%). The median (range) duration of study treatment was 34.3 (0–50.8) months in the D-Rd group and 16.0 (0.2–50.5) months in the Rd group.

### Efficacy

**Fig. 1:**
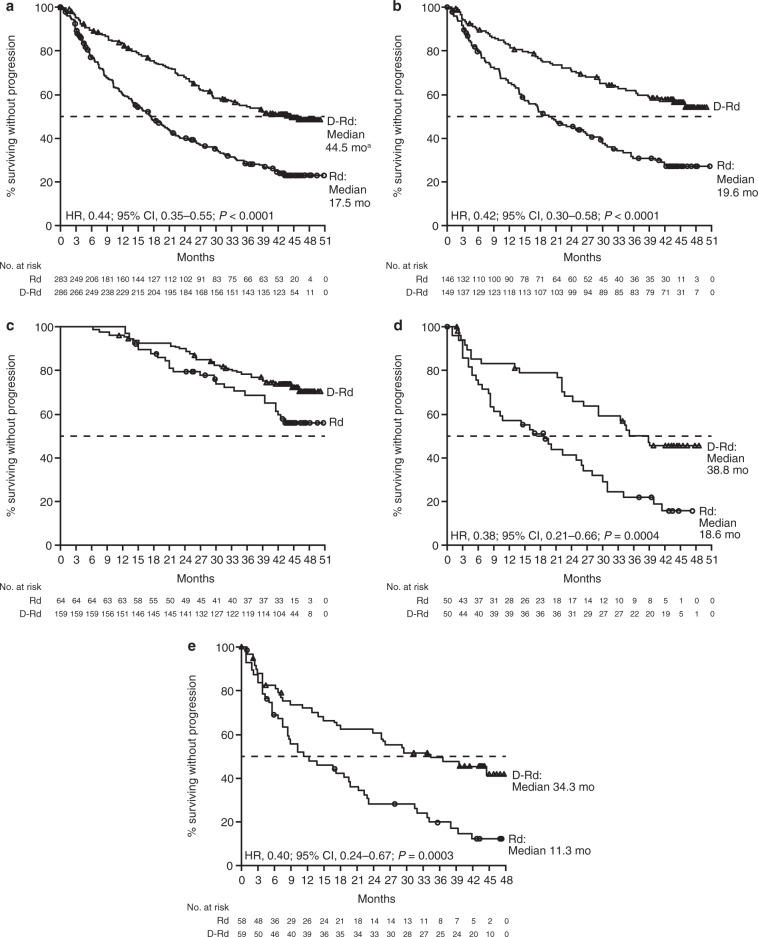
PFS in the ITT population and in patient subgroups based on prior treatment. PFS in (**a**) the ITT population^a^ and in patients with (**b**) one prior line of therapy, (**c**) responses of CR or better, (**d**) prior lenalidomide exposure, or (**e**) refractoriness to bortezomib. Kaplan–Meier estimates of PFS. PFS, progression-free survival; ITT, intent-to-treat; D-Rd, daratumumab/lenalidomide/dexamethasone; Rd, lenalidomide/dexamethasone; HR, hazard ratio; CI, confidence interval; NE, not estimable. ^a^The upper bound of the 95% CI is currently NE.

**Fig. 2:**
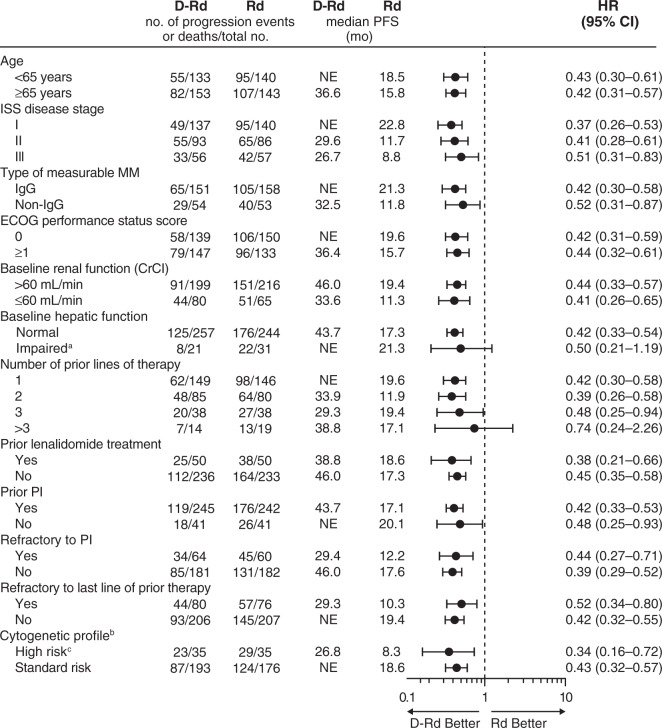
PFS in patient subgroups. PFS, progression-free survival; D-Rd, daratumumab/lenalidomide/dexamethasone; Rd, lenalidomide/dexamethasone; CI, confidence interval; NE, not estimable; ISS, International Staging System; MM, multiple myeloma; Ig, immunoglobulin; ECOG, Eastern Cooperative Oncology Group; CrCl, creatinine clearance; PI, proteasome inhibitor; FISH, fluorescence in situ hybridization. ^a^Impaired hepatic function included mild, moderate, and severe hepatic dysfunction as per National Cancer Institute organ dysfunction criteria. ^b^Cytogenetic risk was determined by FISH or karyotyping. ^c^Patients with high cytogenetic risk had *t*(4;14), *t*(14;16), or del17p abnormalities.

For the primary endpoint, at a median (range) follow-up of 44.3 (0–50.9) months, D-Rd significantly prolonged PFS compared with Rd in the ITT population (median 44.5 [95% CI, 34.1–not estimable] vs 17.5 [95% CI, 13.9–20.8] months; HR, 0.44; 95% CI, 0.35–0.55; *P* *<* 0.0001; Fig. 1a). In the subgroup of patients who received one prior line of therapy, D-Rd (*n* = 149) significantly prolonged PFS versus Rd (*n* = 146; median not reached vs 19.6 months; HR, 0.42; 95% CI, 0.30–0.58; *P* *<* 0.0001; Fig. 1b); 42-month PFS rates were 57.3% versus 27.8%, respectively. Among patients who received one to three prior lines of therapy, D-Rd (*n* = 272) significantly prolonged PFS versus Rd (*n* = 264; median 44.5 vs 17.5 months; HR, 0.43; 95% CI, 0.34–0.54; *P* < 0.0001). In patients who achieved deep responses of CR or better, PFS was prolonged with D-Rd (*n* = 159) versus Rd (*n* = 64), with 42-month PFS rates of 73.6% versus 59.6%, respectively; Fig. 1c. In patients with prior lenalidomide therapy, PFS was significantly prolonged with D-Rd (*n* = 50) versus Rd (*n* = 50; median 38.8 vs 18.6 months; HR, 0.38; 95% CI, 0.21–0.66; *P* *=* 0.0004; Fig. 1d). In the subset of patients with bortezomib-refractory disease, PFS was significantly prolonged with D-Rd (*n* = 59) versus Rd (*n* = 58; median 34.3 vs 11.3 months; HR, 0.40; 95% CI, 0.24–0.67; *P* *=* 0.0003; Fig. 1e). The PFS benefit of D-Rd versus Rd was also maintained in patients who received two or three lines of prior therapy, and in subgroups based on cytogenetic risk status, age, type of MM, ISS disease stage, Eastern Cooperative Oncology Group performance status score, baseline renal or hepatic function, prior treatment exposure, and refractory status (Fig. 2).

**Fig. 3:**
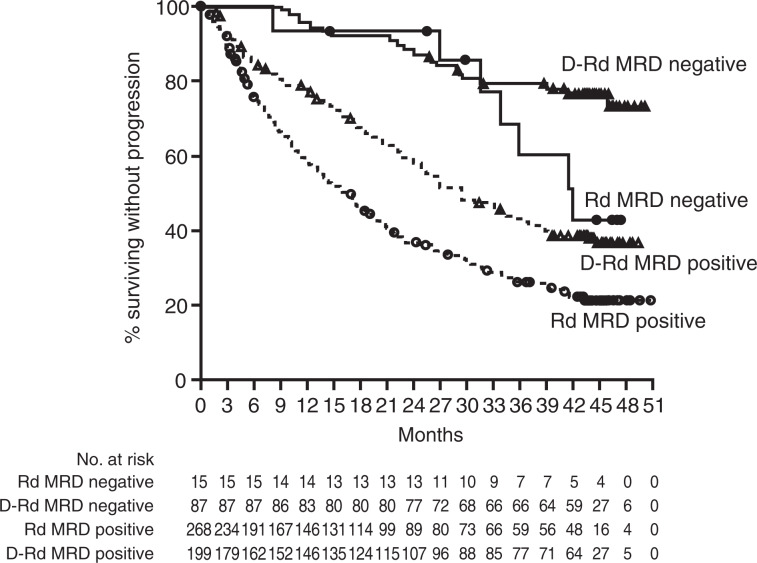
PFS based on MRD status (10^–5^). PFS, progression-free survival; MRD, minimal residual disease; D-Rd, daratumumab/lenalidomide/dexamethasone; Rd, lenalidomide/dexamethasone.

In the response-evaluable population, ORR was significantly higher with D-Rd (*n* = 281) compared with Rd alone (*n* = 276; 92.9 vs 76.4%; *P* *<* 0.0001; Table 1), including higher rates of VGPR or better (80.4 vs 49.3%; *P* *<* 0.0001) and CR or better (56.6 vs 23.2%; *P* *<* 0.0001). Stringent CRs were achieved in 29.2% of patients in the D-Rd group versus 10.5% of patients in the Rd group. Among patients who received prior lenalidomide, ORR was significantly higher with D-Rd (*n* = 50) compared with Rd alone (*n* = 47; 84.0 vs 64.0%; *P* *=* 0.0233; Table 1), including higher rates of VGPR or better (80.0 vs 36.0%; *P* *<* 0.0001), CR or better (54.0 vs 12.0%; *P* *<* 0.0001), and stringent CR (26.0 vs 2.0%). At a sensitivity threshold of 10^−5^, MRD negativity was achieved by 87 (30.4%) patients in the ITT population who received D-Rd versus 15 (5.3%) patients who received Rd (*P* *<* 0.0001). Among patients who achieved MRD negativity (10^−5^), PFS was prolonged with D-Rd versus Rd (median not reached vs 42.0 months; HR, 0.46; 95% CI, 0.19–1.08; *P* = 0.0667; Fig. 3), with 42-month PFS rates of 76.7% with D-Rd and 42.8% with Rd. Among patients with MRD-positive status, D-Rd significantly prolonged PFS compared with Rd (median 29.4 vs 16.0 months; HR, 0.60; 95% CI, 0.48–0.76; *P* < 0.0001; Fig. 3). Among patients who received prior lenalidomide, a similar improvement in the rate of MRD negativity (10^−5^) was observed with D-Rd (32.0%) versus Rd alone (6.0%; *P* = 0.0006).

**Table 1:** Summary of best confirmed response^a^ and MRD-negative^b^ rates.

Variable	D-Rd (*n* = 281)	Rd (*n* = 276)	*P*
Overall response			
No. with response	261	211	
Rate, % (95% CI)	92.9 (89.2–95.6)	76.4 (71.0–81.3)	< 0.0001^c^
Clinical benefit, *n* (%)^d^	266 (94.7)	237 (85.9)	
Best overall response, *n* (%)			
CR or better	159 (56.6)	64 (23.2)	< 0.0001^c^
Stringent CR^e^	82 (29.2)	29 (10.5)	
CR	77 (27.4)	35 (12.7)	
VGPR or better	226 (80.4)	136 (49.3)	< 0.0001^c^
VGPR	67 (23.8)	72 (26.1)	
Partial response	35 (12.5)	75 (27.2)	
Stable disease^f^	18 (6.4)	59 (21.4)	
Progressive disease	0 (0.0)	4 (1.4)	
Response could not be evaluated	2 (0.7)	2 (0.7)	
MRD negative (10^−5^**)**	*n* = 286	*n* = 283	
*n* (%)	87 (30.4)	15 (5.3)	< 0.0001^g^

Response was assessed according to the Uniform Criteria Consensus recommendations of the International Myeloma Working Group [17, 18]. The analysis included patients who had a confirmed diagnosis of MM and measurable disease at baseline or screening. In addition, patients had received at least one administration of trial treatment and had at least one disease assessment after the baseline visit.

*MRD* minimal residual disease, *D-Rd* daratumumab/lenalidomide/dexamethasone, *Rd* lenalidomide/dexamethasone, *CI* confidence interval, *CR* complete response, *VGPR* very good partial response.

^a^Response-evaluable population.

^b^Intent-to-treat population.

^c^*P* value was calculated using the Cochran–Mantel–Haenszel chi-square test.

^d^Clinical benefit includes all patients with minimal response, partial response, VGPR, CR, and stringent CR.

^e^Criteria for a stringent CR include the criteria for a CR plus a normal free light-chain ratio and the absence of clonal plasma cells as assessed by immunohistochemical or immunofluorescence analysis or by flow cytometry.

^f^Includes patients who achieved a minimal response.

^g^*P* value was calculated using the Fisher’s exact test.

**Fig. 4:**
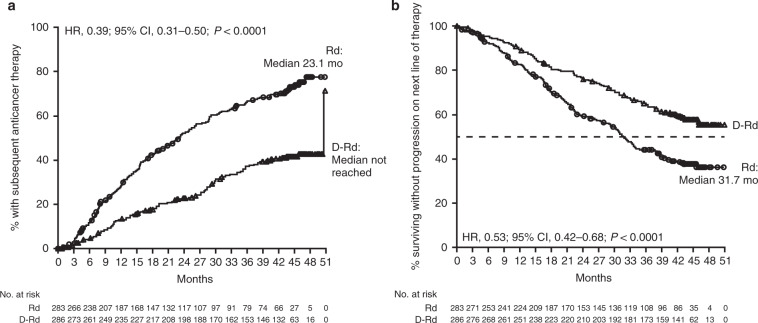
Time to subsequent therapy and PFS2. Time to subsequent therapy (**a**) and PFS2 (**b**) in the ITT population. PFS2, progression-free survival on subsequent line of therapy; HR, hazard ratio; CI, confidence interval; Rd, lenalidomide/dexamethasone; D-Rd, daratumumab/lenalidomide/dexamethasone; ITT, intent-to-treat.

Median duration of response was not reached (95% CI, could not be estimated) with D-Rd compared with 25.2 (95% CI, 19.3–29.7) months with Rd. Median time to next therapy was 50.6 months versus 23.1 months in the D-Rd and Rd arms, respectively (HR, 0.39; 95% CI, 0.31–0.50; *P* *<* 0.0001; Fig. 4a). A total of 63 patients in the Rd group received daratumumab monotherapy after disease progression on Rd. At the time of this analysis, seven patients were on subsequent daratumumab monotherapy. The most common reason for discontinuation of daratumumab monotherapy was disease progression. Median PFS2 was not reached in the D-Rd group versus 31.7 months in the Rd group (HR, 0.53; 95% CI, 0.42–0.68; *P* *<* 0.0001; Fig. 4b), with 42-month PFS2 rates of 59% and 38%, respectively. At the time of this analysis, fewer deaths occurred in patients receiving D-Rd (*n* = 104) compared with Rd (*n* = 121), and median OS was not reached in either group. The 42-month OS rate was 65% with D-Rd versus 57% with Rd. Follow-up for OS is ongoing.

### Safety

No new safety concerns were reported in either treatment group with longer follow-up. The most common treatment-emergent AE (TEAE) was neutropenia, occurring in 63.3% of patients treated with D-Rd and 48.0% of patients who received Rd (Table 2). The most common (≥5%) grade 3/4 TEAEs observed with D-Rd and Rd included neutropenia, febrile neutropenia, anemia, thrombocytopenia, lymphopenia, pneumonia, diarrhea, fatigue, hypokalemia, and cataracts (Table 2). The percentage of patients with TEAEs leading to treatment discontinuation was similar between groups (D-Rd, 14.8; Rd, 14.6%). The most common TEAEs (≥1%) leading to treatment discontinuation with D-Rd versus Rd were pneumonia (1.8 vs 1.1%), pulmonary embolism (0 vs 1.1%), septic shock (1.1 vs 0%), and general physical health deterioration (1.1 vs 0%), respectively. The incidence of second primary malignancies was similar between groups, occurring in 8.5% of patients who received D-Rd and 8.9% of patients who received Rd (Table 3).

**Table 2:** Most common all grade (≥25%) and grade 3/4 (≥5%) TEAEs in the safety population.

Event	D-Rd (*n* = 283)		Rd (*n* = 281)	
	All grade, *n* (%)	Grade 3/4, *n* (%)	All grade, *n* (%)	Grade 3/4, *n* (%)
Total	281 (99.3)	255 (90.1)	274 (97.5)	227 (80.8)
Hematologic				
Neutropenia	179 (63.3)	157 (55.5)	135 (48.0)	117 (41.6)
Febrile neutropenia	18 (6.4)	18 (6.4)	8 (2.8)	8 (2.8)
Anemia	111 (39.2)	50 (17.7)	114 (40.6)	60 (21.4)
Thrombocytopenia	87 (30.7)	42 (14.8)	88 (31.3)	44 (15.7)
Lymphopenia	19 (6.7)	16 (5.7)	17 (6.0)	12 (4.3)
Nonhematologic				
Diarrhea	165 (58.3)	28 (9.9)	105 (37.4)	11 (3.9)
Upper respiratory tract infection	121 (42.8)	5 (1.8)	78 (27.8)	5 (1.8)
Fatigue	110 (38.9)	19 (6.7)	87 (31.0)	12 (4.3)
Cough	99 (35.0)	1 (0.4)	42 (14.9)	0 (0.0)
Nasopharyngitis	96 (33.9)	0 (0.0)	59 (21.0)	0 (0.0)
Constipation	93 (32.9)	3 (1.1)	76 (27.0)	2 (0.7)
Muscle spasms	84 (29.7)	3 (1.1)	60 (21.4)	4 (1.4)
Nausea	82 (29.0)	6 (2.1)	51 (18.1)	2 (0.7)
Insomnia	76 (26.9)	6 (2.1)	63 (22.4)	4 (1.4)
Pyrexia	73 (25.8)	9 (3.2)	40 (14.2)	7 (2.5)
Back pain	71 (25.1)	8 (2.8)	57 (20.3)	5 (1.8)
Pneumonia	71 (25.1)	43 (15.2)	46 (16.4)	28 (10.0)
Edema peripheral	67 (23.7)	2 (0.7)	47 (16.7)	4 (1.4)
Vomiting	62 (21.9)	3 (1.1)	19 (6.8)	4 (1.4)
Dyspnea	61 (21.6)	12 (4.2)	37 (13.2)	2 (0.7)
Bronchitis	57 (20.1)	7 (2.5)	48 (17.1)	9 (3.2)
Asthenia	54 (19.1)	10 (3.5)	46 (16.4)	9 (3.2)
Cataract	54 (19.1)	17 (6.0)	33 (11.7)	12 (4.3)
Hypokalemia	51 (18.0)	17 (6.0)	31 (11.0)	9 (3.2)
Headache	49 (17.3)	0 (0.0)	24 (8.5)	0 (0.0)

*TEAE* treatment-emergent adverse event, *D-Rd* daratumumab/lenalidomide/dexamethasone, *Rd* lenalidomide/dexamethasone.

**Table 3:** Summary of second primary malignancies in the safety population.

	D-Rd (*n* = 283)	Rd (*n* = 281)
Total, *n* (%)	24 (8.5)	25 (8.9)
Cutaneous/noninvasive	12 (4.2)	10 (3.6)
Noncutaneous/invasive	8 (2.8)	11 (3.9)
Hematologic	5 (1.8)	3 (1.1)

*D-Rd* daratumumab/lenalidomide/dexamethasone, *Rd* lenalidomide/dexamethasone.

## Discussion

After >3.5 years of median follow-up, the addition of daratumumab to Rd continued to demonstrate significant clinical benefit over Rd alone in patients with RRMM. At a median follow-up of 44.3 months, D-Rd demonstrated an unprecedented median PFS of 44.5 months versus only 17.5 months for Rd, conferring a 56% reduction in the risk of disease progression or death. At the time of the analysis, the upper bound of the 95% CI for median PFS in the D-Rd group was not estimable. Deep responses, including significantly higher (>5-fold) rates of MRD negativity (10^–5^) were achieved with D-Rd versus Rd alone (30.4 vs 5.3%, respectively), which deepened with longer follow-up [16].

Patients with one prior line of therapy gained the greatest clinical benefit with D-Rd, resulting in a 58% reduction in the risk of disease progression or death compared with Rd. Consistent findings were observed in CASTOR, in which patients who received one prior line of therapy demonstrated the greatest clinical benefit with daratumumab plus bortezomib and dexamethasone (D-Vd) versus bortezomib and dexamethasone (Vd) alone (78% reduction in the risk of disease progression or death), regardless of prior treatment with either lenalidomide or bortezomib [19].

In POLLUX, patients who were refractory to lenalidomide were excluded from the study. However, D-Rd demonstrated improved efficacy outcomes, including prolonged PFS and improved depth of response in patients who received prior lenalidomide but were not refractory to the drug. Furthermore, D-Rd prolonged PFS versus Rd in poor prognostic patient subgroups, including those with ISS stage III disease, patients who were refractory to their last prior line of therapy, and patients with high cytogenetic risk abnormalities, although to a lesser extent in comparison with other patient subgroups evaluated.

Although cross-study comparisons must take into account differences in study population and design, the median PFS observed with D-Rd (44.5 months) is unprecedented in the RRMM treatment setting. In the phase 3 ASPIRE study of carfilzomib plus Rd (KRd) compared with Rd alone in patients with relapsed MM and one to three prior treatments, median PFS was 26.1 versus 16.6 months with KRd and Rd, respectively (HR, 0.66; 95% CI, 0.55–0.78; *P* *<* 0.001), at a median follow-up of 48.8 months for KRd and 48.0 months for Rd [20]. With longer follow-up (median 67.1 months), median OS was 48.3 months for KRd versus 40.4 months for Rd, resulting in 21% reduction in the risk of death (HR, 0.79; 95% CI, 0.67–0.95; *P* = 0.0045) [20]. In the phase 3 TOURMALINE-MM1 study in patients with RRMM and one to three prior therapies, median PFS was 20.6 months with ixazomib in combination with Rd (IRd) versus 14.7 months with Rd alone (HR, 0.74; 95% CI, 0.59–0.94; *P* *=* 0.01), at a median follow-up of 14.8 versus 14.6 months in the IRd and Rd groups, respectively [21]. Median PFS in the phase 3 ELOQUENT-2 study in patients with RRMM and one to three prior therapies was 19.4 months with elotuzumab plus Rd compared with 14.9 months with Rd alone (HR, 0.73; 95% CI, 0.60–0.89; *P* *=* 0.0014), with 3 years of extended follow-up [22].

D-Rd induced deep and durable responses that continued to deepen over time with longer follow-up. At the most recent clinical cutoff, rates of VGPR or better (80.4 vs 49.3%) and CR or better (56.6 vs 23.2%) with D-Rd versus Rd were higher than those observed at primary analysis (VGPR or better: 75.8 vs 44.2%; CR or better: 43.1 vs 19.2%) [12] and at ~2 years of follow-up (VGPR or better: 78.6 vs 47.8%; CR or better: 51.2 vs 21.0%; Supplementary Fig. 1a) [16]. MRD-negative rates have also continued to deepen with D-Rd versus Rd over time (interim analysis: 22.4 vs 4.6% [12]; 2-year follow-up: 26.2 vs 6.4% [16]; current analysis: 30.4 vs 5.3%; Supplementary Fig. 1b), while the rates of MRD negativity with Rd have remained relatively constant with longer follow-up. It is important to note that an updated next-generation sequencing assay with improved calibration rate was used to determine MRD negativity in the current study. PFS was prolonged with D-Rd versus Rd in patients who achieved MRD negativity and significantly prolonged with D-Rd versus Rd in patients with MRD-positive status. The lack of a statistically significant difference in PFS among patients who achieved MRD negativity with D-Rd versus Rd (*P* = 0.0667) may be the result of the low number of patients in the Rd arm (*n* = 15).

PFS2 may serve as a surrogate endpoint for OS when survival data are not available [23]. The use of PFS2 has been suggested as a preferred endpoint, particularly for studies investigating long-term maintenance treatment [23–25]. The findings presented here demonstrate that D-Rd significantly prolongs the time to subsequent therapy and PFS2 versus Rd, conferring a 47% reduction in the risk of disease progression or death on the next line of therapy. These data indicate that treatment with D-Rd does not negatively impact on patient outcomes on subsequent therapy. At the time of this analysis, 17 more deaths occurred with Rd (121/283) compared with D-Rd (104/286). Follow-up for OS in POLLUX is ongoing, with the final analysis planned after 330 deaths.

With longer follow-up, the safety profile of D-Rd and Rd remains largely consistent with the known safety profiles of daratumumab [26] and of Rd [20, 27, 28]. Despite the higher rates of neutropenia and infections (upper respiratory tract infection and pneumonia), the rates of grade 3 or 4 infections were similar between treatment groups and were managed according to local institutional treatment standard-of-care protocols. Consistent tolerability was also observed with daratumumab in the phase 3 MAIA study of D-Rd versus Rd in patients with NDMM who are ineligible for transplantation (median follow-up, 28 months) [15].

Taken together, the results from >3.5 years of median follow-up demonstrate that D-Rd continues to provide significant PFS benefit and induces deeper and more durable responses, including a greater than five-fold increase in the rate of MRD negativity versus Rd alone in patients with RRMM. No new safety concerns were observed following a median of 34 months of D-Rd exposure. These updated findings continue to support the use of D-Rd in patients with RRMM after first relapse.

## Supplementary information

Supplemental Appendix
