# Supplementary material for: Daratumumab plus lenalidomide and dexamethasone in relapsed/refractory multiple myeloma: extended follow-up of POLLUX, a randomized, open-label, phase 3 study
Source: Leukemia. 2020 Jan 30;34(7):1875–84. doi: 10.1038/s41375-020-0711-6 (PMC7326710; doi:10.1038/s41375-020-0711-6)
Supplement: Supplementary file 1 — Supplemental Appendix [file 41375_2020_711_MOESM1_ESM.docx]

**SUPPLEMENTARY APPENDIX**

Supplemental Table 1 presents the baseline patient demographics, prior treatment history, and other clinical characteristics for patients in the intent-to-treat population of POLLUX. Supplemental Figure 1 demonstrates deepening of response rates and the rates of MRD negativity over time with D-Rd.

**Supplemental Table 1. Baseline Demographics and Clinical Characteristics.**

| **Characteristic** | **D-Rd**  **(n = 286)** | **Rd**  **(n = 283)** |
| --- | --- | --- |
| Age, y, n (%) |  |  |
| Median (range) | 65 (34-89) | 65 (42-87) |
| <65 y | 133 (46.5) | 140 (49.5) |
| 65-74 y | 124 (43.4) | 108 (38.2) |
| ≥75 y | 29 (10.1) | 35 (12.4) |
| ISS staging, n (%)^a^ |  |  |
| I | 137 (47.9) | 140 (49.5) |
| II | 93 (32.5) | 86 (30.4) |
| III | 56 (19.6) | 57 (20.1) |
| Type of measureable MM |  |  |
| IgG | 151 (52.8) | 158 (55.8) |
| Non-IgG^b^ | 54 (18.9) | 53 (18.7) |
| ECOG performance score |  |  |
| 0 | 139 (48.6) | 150 (53.0) |
| 1 | 136 (47.6) | 118 (41.7) |
| 2 | 11 (3.8) | 15 (5.3) |
| Baseline renal function (CrCl) |  |  |
| N | 279 | 281 |
| >60 mL/min | 199 (71.3) | 216 (76.9) |
| ≤60 mL/min | 80 (28.7) | 65 (23.1) |
| Baseline hepatic function |  |  |
| N | 278 | 275 |
| Normal | 257 (92.4) | 244 (88.7) |
| Impaired | 21 (7.6) | 31 (11.3) |
| Time from diagnosis, y |  |  |
| Median (range) | 3.48  (0.4-27.0) | 3.95  (0.4-21.7) |
| Prior lines of therapy, n (%) |  |  |
| Median (range) | 1 (1-11) | 1 (1-8) |
| 1 | 149 (52.1) | 146 (51.6) |
| 2 | 85 (29.7) | 80 (28.3) |
| 3 | 38 (13.3) | 38 (13.4) |
| >3 | 14 (4.9) | 19 (6.7) |
| Prior lenalidomide |  |  |
| Yes | 50 (17.5) | 50 (17.7) |
| No | 236 (82.5) | 233 (81.5) |
| Prior PI |  |  |
| Yes | 245 (85.7) | 242 (85.5) |
| No | 41 (14.3) | 41 (14.5) |
| Refractory to PI^c^ |  |  |
| Yes | 64 (22.4) | 60 (21.2) |
| No | 181 (63.3) | 182 (64.3) |
| Refractory to last line of prior therapy |  |  |
| Yes | 80 (28.0) | 76 (26.9) |
| No | 206 (72.0) | 207 (73.1) |
| Cytogenetic profile^d^ |  |  |
| N | 228 | 211 |
| Standard risk | 193 (84.6) | 176 (83.4) |
| High risk^e^ | 35 (15.4) | 35 (16.6) |

D-Rd, daratumumab/lenalidomide/dexamethasone; Rd, lenalidomide/dexamethasone; ISS, International Staging System; MM, multiple myeloma; IgG, immunoglobulin G; ECOG, Eastern Cooperative Oncology Group; CrCl, creatinine clearance; PI, proteasome inhibitor; FISH, fluorescence in situ hybridization.

^a^ISS staging is derived based on the combination of serum β2-microglobulin and albumin.

^b^Includes IgA, IgD, IgM, IgE, and biclonal.

^c^Refractory to PI subgroup analysis is based on patients who received prior PI therapy.

^d^Based on FISH or karyotype testing.

^e^Patients with high cytogenetic risk had a t(4;14), t(14;16), or del17p abnormality.

**Supplemental Figure 1. Response rates over time (A) and time to MRD negativity (10^–5^; B).**

(A) Response rates were evaluated in the response-evaluable population. (B) Time to initial MRD negativity at a sensitivity threshold of 10^–5^ was evaluated in the intent-to-treat population.


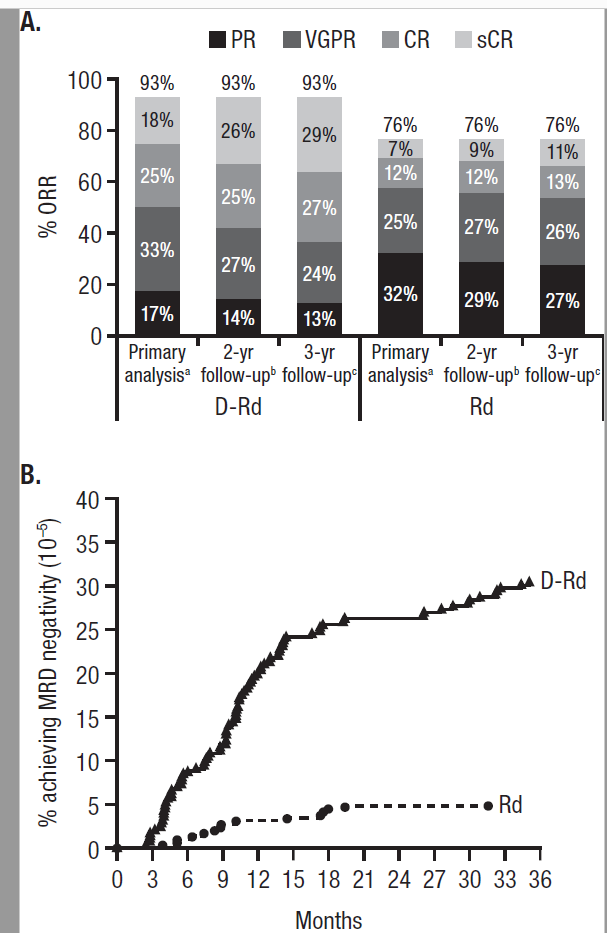


MRD, minimal residual disease; PR, partial response; VGPR, very good partial response; CR, complete response; sCR, stringent complete response; ORR, overall response rate; D-Rd, daratumumab/lenalidomide/dexamethasone; Rd, lenalidomide/dexamethasone.

^a^Median follow-up 13.5 months.
^b^Median follow-up 25.4 months.
^c^Median follow-up 44.3 months.
